# Emergence of Flexible White Organic Light-Emitting Diodes

**DOI:** 10.3390/polym11020384

**Published:** 2019-02-22

**Authors:** Dongxiang Luo, Qizan Chen, Baiquan Liu, Ying Qiu

**Affiliations:** 1 School of Materials and Energy, Guangdong University of Technology, Guangzhou 510006, China; luodx@gdut.edu.cn (D.L.); 18219435079@163.com (Q.C.); 2 LUMINOUS! Centre of Excellent for Semiconductor Lighting and Displays, School of Electrical and Electronic Engineering, Nanyang Technological University, Nanyang Avenue, Singapore 639798, Singapore; 3 Institute of Polymer Optoelectronic Materials and Devices, State Key Laboratory of Luminescent Materials and Devices, South China University of Technology, Guangzhou 510640, China; 4 Guangdong R&D Center for Technological Economy, Guangzhou 510000, China

**Keywords:** flexible, OLED, white, high efficiency, exciton

## Abstract

Flexible white organic light-emitting diodes (FWOLEDs) have considerable potential to meet the rapidly growing requirements of display and lighting commercialization. To achieve high-performance FWOLEDs, (i) the selection of effective flexible substrates, (ii) the use of transparent conducting electrodes, (iii) the introduction of efficient device architectures, and iv) the exploitation of advanced outcoupling techniques are necessary. In this review, recent state-of-the-art strategies to develop FWOLEDs have been summarized. Firstly, the fundamental concepts of FWOLEDs have been described. Then, the primary approaches to realize FWOLEDs have been introduced. Particularly, the effects of flexible substrates, conducting electrodes, device architectures, and outcoupling techniques in FWOLEDs have been comprehensively highlighted. Finally, issues and ways to further enhance the performance of FWOLEDs have been briefly clarified.

## 1. Introduction

Organic light-emitting diodes (OLEDs) are now entering the mainstream display and lighting market thanks to their exceptional merits such as high efficiency, low power consumption, fast response, and their outstanding compatibility with flexible substrates [1,2,3,4,5]. Since the first OLED invented by Tang et al. in 1987 [6], the performance of OLEDs (e.g., external quantum efficiency (EQE), current efficiency (CE), power efficiency (PE), luminance or brightness, lifetime, and voltage) have been greatly enhanced [7,8,9,10,11]. Currently, it is believed that OLED technology will not only dominate the next-generation displays, but also promises to be comparable to inorganic GaN-based LEDs in the field of lighting. In addition, by dint of the reported concepts in OLEDs, many other related optoelectronic techniques have been rapidly developed [12,13,14,15]. In particular, various types of LEDs are thriving such as polymer LEDs [16,17,18,19], colloidal quantum-dot LEDs [20,21,22,23], perovskite LEDs [24,25,26,27], and colloidal quantum-well LEDs [28,29,30]. Thus, the further enhancement of OLED technology is also beneficial to the optoelectronic fields.

To render OLEDs more competitive than other display and lighting counterparts, flexible OLEDs have steadily attracted both scientific and industrial interest owing to their unique merits, including ultralight weight, small thickness, and suitability for roll-to-roll production [31,32,33,34,35]. Compared with conventional OLEDs, which are mostly manufactured on rigid glass substrates, flexible OLEDs offer the possibility of enduring vast mechanical deformation (e.g., rolling, stretching, rotating, curving, folding, twisting, or more complicated appearances) [36,37,38,39,40]. Therefore, flexible OLEDs can make our life more artistic (e.g., flexible OLEDs can be designed to apply in bags, foldable phones, curved lamps, and so on). In 1992, Heeger et al. took the first step to fabricate a flexible OLED with soluble conducting polymers on a polyethylene terephthalate (PET) substrate [41]. In 1997, Forrest et al. established the first flexible OLED with small-molecule organic materials [42]. Since then, a large amount of attention has been paid to pursuing flexible OLEDs [43]. With excellent results, Lu et al. used an efficient anode stack and a lens-based structure to unlock the full potential of a green OLED on flexible plastic, achieving a maximum PE of 290 lm W^−1^ [44]. Nowadays, commercial flexible OLED smartphones emerge in the market [45]. 

In general, white OLEDs (WOLEDs) are desirable to high-quality displays and solid-state lighting [46,47,48,49,50,51]. By carefully designing a single molecule emitter, and using complementary-color, three-color, or four-color emitters (e. g., blue/yellow, blue/green/red, blue/green/yellow/red), a great number of WOLEDs have been constructed [52,53,54,55,56]. In the case of traditional WOLEDs based on glass substrates, the PE has been increased from the initial value of 0.83 lm W^−1^ [57,58] to the current value of 123 lm W^−1^ at 1000 cd m^−2^ (EQE: 54.6%) in the literature [59]. For other parameters of traditional WOLEDs (e.g., lifetime [60,61], color rendering index (CRI) [62,63,64,65,66], correlated color temperature (CCT) [67,68,69], luminance [70,71,72], and color stability [73,74,75,76]), they have also been demonstrated to satisfy the demand of real commercialization. In terms of flexible WOLEDs (FWOLEDs), growing efforts have been taken in recent years due to the booming demand for consumer electronics. In 2005, Mikami et al. reported the first FWOLED with a PE of 4.3 lm W^−1^ [77]. Although the efficiency is not high due to the use of fluorescent emitters, Mikami’s work starts the step towards FWOLEDs. By employing phosphorescent or thermally activated delayed fluorescence (TADF) emitters, the efficiency of FWOLEDs can be boosted [78,79,80,81,82]. This is because both phosphors and TADF materials can realize a theoretical unity internal quantum efficiency (IQE) via the heavy-atom effect [83,84,85,86,87] and small energy gap (ΔE_ST_) between triplet excited state (T_1_) and singlet excited state (S_1_) [88,89,90,91,92], respectively. Hence, both singlet and triplet excitons will be harvested. Keeping these facts in mind, the efficiency of FWOLEDs (e.g., a maximum EQE of 72.4% and PE of 168.5 lm W^-2^) can be as high as that of traditional WOLEDs based on glass substrates [93]. In addition, the CRI, Commission International de L’Eclairage (CIE) chromaticity coordinates and other parameters of FWOLEDs have been step-by-step improved. Furthermore, apart from bottom-emitting FWOLEDs, top-emitting FWOLEDs and transparent FWOLEDs have been demonstrated [94,95,96]. As a result, these structure innovations provide FWOLEDs with the ability to meet the demand of various applications.

Herein, we will summarize recent state-of-the-art strategies to develop FWOLEDs. Firstly, we will describe the fundamental concepts of flexible OLEDs. Then, we will introduce the main strategies to realize FWOLEDs. Particularly, we will comprehensively highlight the effects of flexible substrates, conducting electrodes, device architectures, and outcoupling techniques in FWOLEDs, which are expected to give a deep understanding of developing FWOLEDs. Finally, we will briefly clarify issues and ways to further enhance the performance of FWOLEDs.

## 2. Fundamental Concepts of FWOLEDs

### 2.1. Flexible Substrates

**Figure 1:**
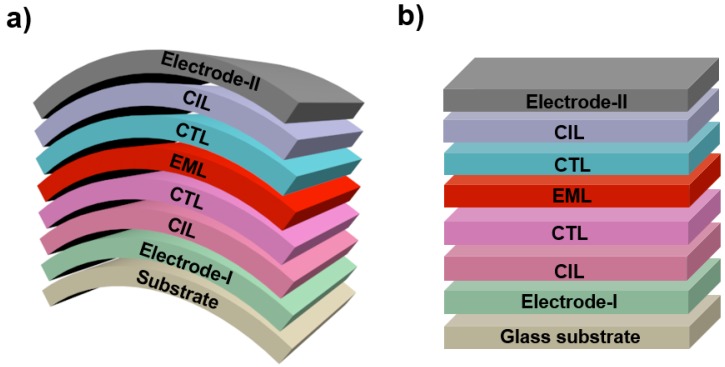
Diagram of the device structure of flexible white organic light-emitting diodes (FWOLEDs) (**a**) and conventional white organic light-emitting diodes (WOLEDs) based on rigid glass substrates (**b**). CIL is the charge injection layer, EML is the emitting layer, and CTL is the charge transport layer.

The biggest difference between conventional WOLEDs based on rigid glasses and FWOLEDs is the use of flexible substrates [97,98,99,100,101]. To construct a FWOLED, a flexible substrate is first selected. Then, the bottom electrode is patterned (electrode-I), as shown in Figure 1a. For different applications, electrode-I should be accordingly adjusted (e.g., electrode-I is transparent for bottom-emitting and transparent FWOLEDs but can be opague for top-emitting FWOLEDs). Subsequently, the first charge injection and transport layer, emitting layers (EMLs), the second charge injection and transport layer and the top electrode (electrode-II) are deposited. The role of charge injection and transport layers in FWOLEDs is to facilitate holes or electrons to arrive at the EMLs [102,103,104,105,106], which is similar to that of conventional WOLEDs (Figure 1b). For electrode-II, opaque and transparent metals are usually adopted for bottom-emitting FWOLEDs and top-emitting/transparent FWOLEDs, respectively. Unlike conventional WOLEDs, the electrodes, charge injection and transport layers, and EMLs in FWOLEDs will undergo mechanical deformation if the substrate is bent or stretched. As a consequence, current leakage occurs more easily in FWOLEDs compared with that of conventional WOLEDs [107,108,109,110,111]. Hence, flexible electrodes should be well designed. 

So far, several types of flexible substrates have been reported (Table 1), such as metal foils, very thin glass plastics, polydimethylsiloxane, rubber, and silk [112,113,114,115,116]. To be an excellent candidate material for flexible substrates, some requirements should be satisfied including remarkable mechanical deforming ability, smooth surface, good thermal durability, high oxygen and moisture blocking capability, and effective transparency for bottom-emitting and transparent FWOLEDs [117,118,119]. Among flexible substrates, although metal foils can undergo high working temperatures, the relatively heavy weight, rough surface, and insulating layers located between substrates and devices complicate FWOLEDs. Additionally, the intrinsic opaque property of metal foils restricts the applications in bottom-emitting and transparent FWOLEDs. In the case of thin glasses, despite good thermal durability, the brittle and fragile characteristics hinder the further development of flexible glass substrates. Therefore, considering the trade-off between cost and performance, plastics-based flexible substrates have been aggressively explored, although thermal durability is not good enough. For example, PET, polyethylene naphthalate (PEN), polyimide (PI), and polyethersulfone (PES) are the most widely adopted flexible plastic substrates for LEDs [120,121,122,123].

### 2.2. Conducting Electrodes

For bottom-emitting and transparent FWOLEDs, electrode-I should be of outstanding optical transparency (e.g., a transmittance of >90% in the visible regime), good electrical conductivity (e.g., a sheet resistance of <20 Ω sq^−1^), and mechanical flexibility. To date, indium tin oxide (ITO) is the most commonly used electrode. However, the mechanical flexibility of ITO is not satisfactory enough. Thus, a large number of efforts have been made to replace ITO with metal nanowires or grids, carbon-based materials (e.g., graphene, carbon nanotubes, reduced graphene oxide), conducting polymers, and so on [124,125,126,127,128]. In particular, the transmittance of graphene films greatly rely on the crystal quality. As an excellent result, a transmittance of >97.7% can be achieved for monolayer graphene [97]. In addition, because of the low sheet resistance, high transparency, high flexibility, and the low temperature solution-processed fabrication processes, metal nanowires are promising to be electrode-I [129]. Particularly, silver nanowire is the most broadly employed metal nanowire, since it can form a high-quality ink by uniformly dispersing into ethanol. In addition, silver nanowires can break the waveguide mode and the total internal reflection, enhancing the outcoupling efficiency [130,131,132]. However, the cost and availability of silver means that it is not ideal for widespread commercialization. As an alternative, attention has been paid to copper nanowires, because cooper has a relatively low price and possesses high conductivity next to silver (e.g., copper is 1000 times more abundant and the cost is only 1% compared with silver) [133,134,135,136]. For metal grids, silver and copper are also the most widely exploited elements. In addition, carbon-based materials have been intensively investigated owing to the ease of fabrication and low cost. However, the resistance of carbon-based materials is relatively high, presenting a challenge for the applications. In terms of conducting polymers (e.g., the well-known poly(3,4-ethylenedioxythiophene)/polystyrenesulfonate (PEDOT:PSS)), the inherently limited conductivity is an obstacle for the further development [137]. 

On the other hand, in terms of top-emitting FWOLEDs, electrode-I should exhibit high reflectivity when it is deposited on thin glass or plastic substrates. Owing to the high electrical conductivity and reflectivity, silver has been extensively utilized as the anode in top-emitting devices [138]. However, the work function of silver is very low (4.3 eV). Hence, interface modifying layers (e.g., molybdenum oxide, 1, 4, 5, 8, 9, 11-hexaazatriphenylene hexacarbonitrile (HAT-CN), p-doping technique) are usually introduced to reduce the hole barrier between the anode and hole transport layer, lowering the voltages [139]. For metal foil based flexible substrates, the highly reflective distributed Bragg reflector (DBR) has been reported to be a good candidate for the substrate insulating layer for top-emitting OLEDs [140]. In addition, many composite electrodes have also been explored such as an AuCl_3_-modied graphene electrode [141], silver-nanoparticles modified graphite electrode [142], ZnO/Ag/ZnO nanofilm electrode [143], and so on [144,145,146,147,148,149].

### 2.3. Device Architectures

To guarantee the high-performance of FWOLEDs, effective device architectures are needed. Owing to the effect of spin statistics, singlets and triplets will be formed with a ratio of 1:3 upon hole meeting electrons [150,151,152,153,154]. For fluorophors, singlets rapidly decay with the prompt nanoseconds fluorescence, while the radiative triplets decay is spin forbidden [155,156,157,158]. Thus, the maximum IQE of fluorescent WOLEDs is only 25%. By selecting phosphors or TADF emitters, the IQE of WOLEDs can be as high as 100% due to the harvest of both singlets and triplets [159,160,161,162]. According to the employed emitters, WOLEDs can be classified into four types, i.e., fluorescent WOLEDs, phosphorescent WOLEDs, TADF WOLEDs, and the so-called fluorescent/phosphorescent hybrid WOLEDs [163,164,165,166,167].

After the selection of emitters, the manipulation of charge and exciton distribution is key to achieve high performance [168,169,170,171,172]. This is because each process of charge injection, charge transport, exciton generation, exciton recombination, exciton radiative or nonradiative decay, exciton diffusion, exciton harvest, and energy transfer will affect the device performance [173,174,175,176,177]. As a matter of fact, a great plenty of efficient device design concepts have been reported to manipulate the charge and exciton distribution. For example, Zhu et al. designed a smart design of the emissive zone structure to make full use of generated excitons, realizing a phosphorescent WOLED with >20% EQE [178]. Sun et al. used 4,4′-N,N′-dicarbazole-biphenyl (CBP) as the host and interlayer to harness all excitons, demonstrating a hybrid WOLED with an EQE of 21.2% [179]. By precisely allocating excitons, TADF WOLEDs with >20% EQE have also been developed [180]. In particular, since a blue fluorescent emitter cannot harvest triplets, the charge and exciton distribution should be carefully manipulated in hybrid WOLEDs, otherwise exciton quenching will occur between fluorophors and phosphors [181,182,183,184,185]. To overcome these obstacles, the introduction of interlayer or the adoption of blue fluorophor with high T_1_ is necessary [186,187,188,189,190]. By applying the efficient device architectures to FWOLEDs, high performance can be expected.

### 2.4. Outcoupling Technologies

**Figure 2:**
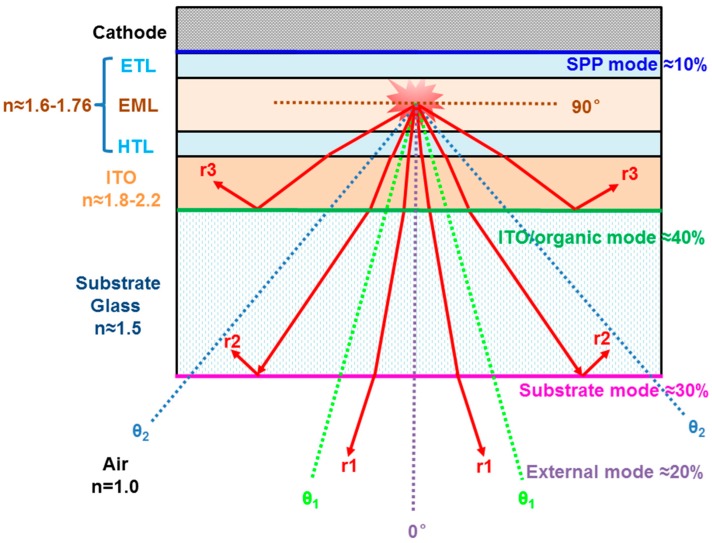
Schematic process of ray propagation for the planar OLED. n is the refractive index, ETL is electron transport layer, HTL is hole transport layer. r1, r2, and r3 represent the rays propagating in the external mode (0° ≤ θ < θ_1_), the substrate mode (θ_1_ ≤ θ < θ_2_), and the indium tin oxide (ITO)/organic mode (θ_2_ ≤ θ < 90°), respectively.

By virtue of phosphorescent or TADF emitters, the IQE of WOLEDs can be as high as 100%. However, the EQE (*η_ext_*) is also decided by the outcoupling factor, which can be defined as [155,191]:

(1) $$\eta_{ext}=\eta_{out}\cdot r\cdot q\cdot \gamma ,$$

where *η_out_* is the outcoupling factor, *r* is the fraction of excitons that can potentially radiatively decay, *q* is the photoluminescence quantum efficiency of the emitter, and *γ* is the charge balance. From this equation, it is easily seen that the EQE is directly proportional to the outcoupling factor. In addition, PE can be described as:

(2) $$\mathrm{PE}\propto \frac{\eta_{out}\cdot r\cdot q\cdot \gamma }{U},$$

where *U* is the voltage. Therefore, the means of enhancing the light extractive efficiency is crucial to the further improvement of EQE. Due to the wave-guiding effect, which results from the mismatch of refractive index (n) among organic layers (n ≈ 1.6–1.76), transparent electrodes (e.g., n ≈ 1.8–2.2 for ITO), glass substrate (n ≈ 1.5), and air (n ≈ 1.0), most photons generated by exciton recombination are trapped via the total internal reflection at interfaces inside conventional bottom-emitting OLEDs [192,193,194], as shown in Figure 2. According to classical ray optics theory (i.e., Snell’s law), the substrate waveguide mode from the total internal reflection at the interface of glass/air and the ITO/organic waveguide mode lead to the fact that the theoretical limit for the EQE of conventional bottom-emitting OLEDs remains near 20%, as described by the below equation [195]:

(3) $$\eta_{out}=\frac{1}{\xi n^{2}},$$

where *n* is the refractive index of organic material (e.g., taking the value of 1.6) and *ξ* is a constant that depends on the dipole alignment and the geometry of the OLED device (e.g., taking the value of 2), and the isotropic emission in the organic layer and perfectly reflecting cathode are assumed. However, it is worth noting that the surface plasmon-polariton (SPP) mode associated with metallic electrode/organic interface, which is not considered by Equation (2), is also an enormous optical loss [196,197,198]. According to classical Snell’s law with refractive indexes of air, substrate, and organic layer, the critical angle at the interface of different layers can be calculated by:

*n*_1_ × *sinθ*_1_ = *n*_2_ × *sinθ*_2_, (4)

where n_1_ and n_2_ are the refractive indexes of adjacent layers and θ_1_ and θ_2_ are the critical angle at air/substrate and substrate/ITO interfaces, respectively. When the light is propagated from the optically thinner medium to optically denser medium, the light will not be reflected at the interface. Hence, θ_1_ and θ_2_ are independent of the ITO since its refractive index is higher than that of the organic layer. Apart from the SPP mode (optical loss ≈10%), the generated light suffers from further three modes: (i) the external mode (≈20%), where the light is emitted from OLEDs (0° ≤ θ < θ_1_); (ii) the substrate mode (optical loss ≈30%), where the light is trapped in the substrate at the glass/air interface due to the total internal reflection and usually propagates to the edge of the glass (θ_1_ ≤ θ < θ_2_); and (iii) the ITO/organic mode (optical loss ≈40%), where the light is trapped at the ITO/substrate interface due to the total internal reflection and dissipated by the ITO, organic, and metal cathode layers (θ_2_ ≤ θ < 90°). Particularly, if ITO is replaced with metallic electrodes (e.g., thin metal film, metal nanowire or grid), the ITO/organic waveguide mode will be eliminated. In such case, the SPP mode is a significant optical loss, especially for OLEDs with two metal electrodes since the SPP mode exists at the anode/organic interface and the cathode/organic interface.

In flexible OLEDs, the total internal reflection will also occur at interfaces due to the difference of refractive index [199,200,201]. However, for the most popular plastic substrates, their refractive index (e.g., ~1.65 for PET) is higher than that of glass. Hence, the external mode, substrate mode, and ITO/organic mode are different from conventional OLEDs with glass substrates, although the SPP mode remains unchanged [202]. By using plastic substrates, the ITO/organic mode emission can be redistributed to the substrate mode. Ideally, this redistribution will completely occur if the refractive index of flexible substrates is equal to that of ITO. Then, external outcoupling technologies (e.g., scattering layers, microlens arrays) can be used to extract the substrate mode emission to the external mode, enhancing the EQE. Aside from the external outcoupling technologies, many internal outcoupling technologies (e.g., photonic crystals, microcavity structures, periodic diffraction gratings, nanoimprinted quasi-random photonic structures) have also been reported [203,204,205,206,207].

## 3. Strategies to Achieve FWOLEDs

### 3.1. Basic Aspects of FWOLEDs

According to the above concepts, four factors are critical to the performance of FWOLEDs, i.e., flexible substrates, conducting electrodes, device architectures and outcoupling techniques. In particular, the substrates and electrodes in FWOLEDs are unlike conventional WOLEDs based on glass substrates. Hence, many attempts have been made to resolve the issues regarding these two factors, especially flexible electrodes. For the design of device structures in FWOLEDs, the concepts in conventional WOLEDs can be used (i.e., the selection of phosphorescent or TADF emitters is essential to harvest the triplet excitons, and the management of charge and exciton distribution is key to the performance). In the case of outcoupling techniques, many approaches in conventional WOLEDs can be adopted for reference, particularly for the external outcoupling techniques. By carefully considering the four factors, high-performance FWOLEDs can be expected. Given that conventional WOLEDs are usually classified into four types according to the employed emitters (i.e., fluorescent, phosphorescent, TADF, and hybrid WOLEDs) [208,209,210,211,212,213,214,215,216,217], FWOLEDs can also be simply assorted via these types. In the following sections, the main strategies to develop various kinds of FWOLEDs have been highlighted from the used substrates, electrodes, and device structures to outcoupling schemes.

### 3.2. FWOLEDs Based on Fluorescent Emitters

#### 3.2.1. Fluorescent FWOLEDs with Indium-Zinc-Oxide Anode

At the initial stage of the development of FWOLEDs, fluorescent emitters were usually used, leading to the waste of triplet excitons. In addition, only little attention has been paid to the outcoupling techniques. Hence, the performance of FWOLEDs is not impressive. However, compared with conventional fluorescent WOLEDs prepared on glasses, fluorescent FWOLEDs can exhibit higher efficiency due to the high-refractive-index plastic substrate which eliminates the optical loss and converts the anode/organic mode into the substrate mode. Significantly, such strategy makes a step forward in the evolution of FWOLEDs.

**Figure 3:**
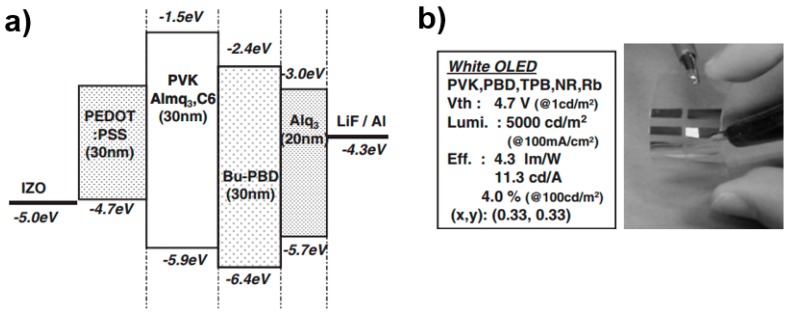
(**a**) Device structure and potential energy diagram. (**b**) The performance of the FWOLED and a photograph for the working FWOLED [77]. Copyright (2005) with permission from IOP Publishing.

In 2005, Mikami et al. realized the fluorescent bottom-emitting FWOLED [77]. The flexible substrate was a heat-resistant (>200 °C) and high-refractive-index (n = 1.65) plastic base film with a protective layer and a thin inorganic gas barrier. The anode was the transparent indium-zinc-oxide (IZO), which was similar to ITO. As shown in Figure 3, the device structure was IZO/PEDOT:PSS/EML/2-(4-Biphenylyl)-5-(4-tert-butylphenyl-1,3,4-oxadiazole) (Bu-PBD, hole-blocking layer)/8-hydrooxyquinoline aluminum-salt (Alq_3_, electron transport layer)/LiF/Al (cathode), where the EML was poly-(N-vinylcarbazole) (PVK): 1,1,4,4-tetraphenyl-1,3-butadiene (TPB): nile red for a FWOLED with an EQE of 2%. To improve the efficiency, 0.23 wt % rubrene was codoped into PVK, assisting the red emission. This was because the excitation energy could be transferred to rubrene from Bu-PBD with a subsequent transfer to nile red. As a result, the FWOLED showed an EQE and PE of 4.0% and 4.3 lm W^−1^ at 100 cd m^−2^, respectively. The efficiency was improved by a factor of 10–20% compared with that of normal glass (n = 1:52), since the IZO/organic mode was reduced by a factor of 46% when the normal glass substrate was replaced with a high-refractive-index (n = 1:65) plastic substrate. Therefore, the key features to enhance the efficiency could be summarized: (i) the improvement of device engineering, and (ii) the utilization of high-refractive-index plastic substrate.

#### 3.2.2. Fluorescent FWOLEDs Using Modified ITO Anode

If transparent conductive oxides (e.g., IZO, ITO) are functioned as the electrode on plastic substrates, the electrical conductivity may not be good enough. This is because plastics usually have a low glass transition temperature and a soft shape, which prevents transparent conductive oxides from being formed at high temperatures and making the surface smooth. 

**Figure 4:**
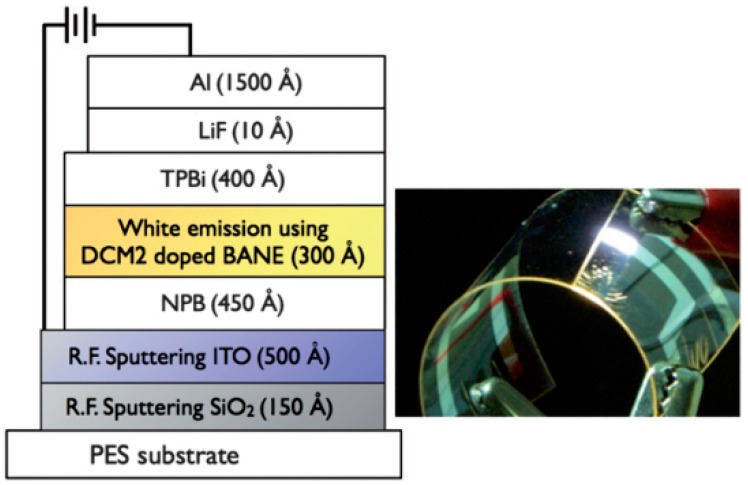
A schematic illustration of the structure of the FWOLED. The inset shows the photograph of the resultant device at emission under bending [218]. Copyright (2010) with permission from Royal Society of Chemistry.

To enhance the electrical conductivity, Jou et al. built a FWOLED by utilizing effective device structure on high glass-transition plastic substrate with a thin silicon dioxide (SiO_2_) pre-coat and ITO deposited using radio frequency magnetron sputtering at elevated temperature [218]. More specifically, PES was used as the flexible substrate (Figure 4). SiO_2_, with the optimized thickness of 15 nm, was introduced on top between the ITO and PES substrate, improving the interfacial adhesion and enhancing resistance to the diffusion of moisture. Then, ITO was obtained using radio frequency magnetron sputtering at 200 °C. After this, a WOLED structure of *N,N′*-bis-(1-naphthy)-N,N′diphenyl-1,1′-biphenyl-4-4′-di-amine (NPB, 45 nm, an electron-blocking and hole-transport layer)/EML (30 nm)/2,2′,2″-(1,3,5-benzenetriyl)-tris(1-phenyl-1-H-benzimidazole (TPBi, 40 nm, a hole-blocking and electron-transport layer)/LiF (1 nm)/Al (150 nm) was proposed, where the EML was 10,10′-di(biphen-4-yl)-9,9′-bianthracene (BANE): 0.05 wt % red dye 4-(dicyanomethylene)-2-methyl-6-(julolidin-4-ylethenyl)-4H-pyran (DCM2) in a single EML. As a result, the FWOLED showed a PE of 6.5 lm W^−1^ at 800 cd m^−2^ and a maximum EQE of 3.2% with a pure-white light with chromaticity coordinates (0.321, 0.339). The key features to realize such a FWOLED could be summarized: (i) the appropriate sputtering temperature of 200 °C for ITO ensured the pure-white emission and high electrical conductivity (e.g., the emission changes markedly from pure-white to bluish-white for ITO sputtered at 200 °C or above, the ITO films sputtered at 200 °C exhibited a much higher conductivity, more than two times that at room temperature); (ii) the optimized thickness of SiO_2_ guaranteed that the ITO film deposited on PES showed a smooth surface with a roughness of 1.4 ± 0.01 nm and a low resistivity of 3.9 × 10^−4^ Ω cm, enhancing the efficiency.

#### 3.2.3. Fluorescent FWOLEDs Exploiting a Four-Layer Graphene Anode

To replace the most widely used ITO anode, lots of attention has been paid to the graphene anode. This is because graphene is a flexible two-dimensional sheet of *sp*^2^-hybridized carbon atoms, which can lower the cost and overcome the brittle issue of ITO. Before 2012, the OLEDs based on graphene anodes usually showed poor performance, since graphene films have a low work function (~4.4 eV) and high sheet resistance (>300 Ω sq^−1^) [219,220].

**Figure 5:**
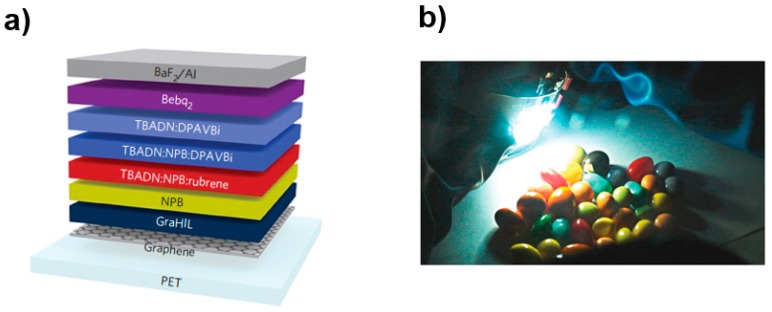
(**a**) Device structure of FWOLED with a graphene anode. (**b**) FWOLED lighting device with a graphene anode on a 5 cm × 5 cm polyethylene terephthalate (PET) substrate [221]. Copyright (2012) with permission from Springer Nature.

To overcome this problem, Han et al. developed a fluorescent FWOLED based on a four-layer graphene anode with a high work function (5.95 eV) and a low sheet resistance (~30 Ω sq^−1^) [221]. The key features of such modified graphene anode could be summarized: (i) graphene films were p-doped with HNO_3_ or AuCl_3_, decreasing the sheet resistance; (ii) a work function gradient from the graphene to the overlying organic layer was created by using conducting polymer compositions to modify the surface (i.e., a self-organized gradient hole injection layer composed of PEDOT:PSS and tetrafluoroethylene-perfluoro-3,6-dioxa-4-methyl-7-octenesulphonic acid copolymer, one of the perfluorinated ionomers (PFI)), which enabled holes to be injected easily into the organic layer. Then, by using a flexible PET substrate and an efficient device architecture, a FWOLED was constructed with the EML of the dopants (skyblue-emitting 4,4′-bis[2-{4-(N,N-diphenylamino)phenyl}vinyl] (DPAVBi) and orange-red-emitting 5,6,11,12-tetraphenylnaphthacene (rubrene)) doping into separate layers in the 2-(tertbutyl)-9,10-bis(2′-naphthyl)anthracene (TBADN) host, as shown in Figure 5. As a result, the first FWOLED based on a graphene anode was developed with a maximum CE of 16.3 cd A^−1^, which was higher than that of the ITO-based WOLED (10.9 cd A^−1^). 

### 3.3. FWOLEDs Based on Hybrid Emitters

Unlike bottom-emitting FWOLEDs, the light of top-emitting FWOLEDs is emitted from the top side. Hence, top-emitting FWOLEDs can be fabricated on opaque substrates (e.g., metal foils), in addition to thin glass and plastics. A main challenge of developing high-performance FWOLEDs is how to reduce the effect of microcavity, which is induced by the two electrodes. Especially, top-emitting FWOLEDs with high CRI are hardly achieved. To eliminate the microcavity effect, the top electrode should be as transparent as possible.

**Figure 6:**
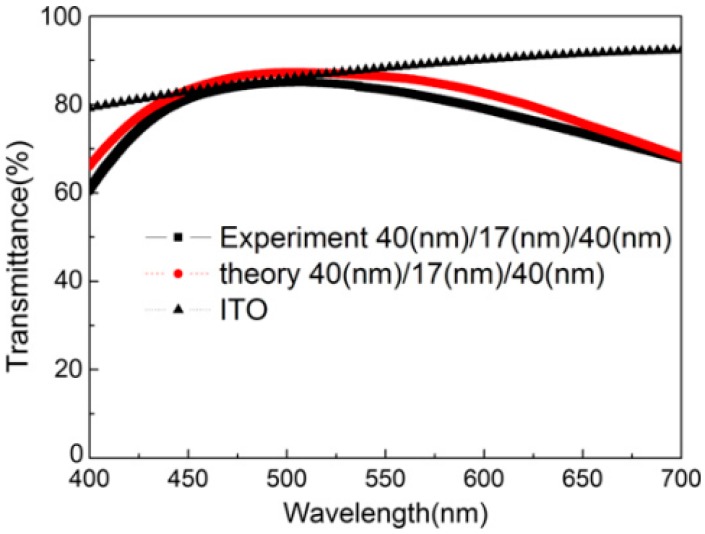
The measured optical transmittance curves of MoO_3_ (40 nm)/Ag (17 nm)/MoO_3_ (40 nm) and ITO, the calculated optical transmittance of MoO_3_ (40 nm)/Ag (17 nm)/MoO_3_ (40 nm) was also plotted [222]. Copyright (2011) with permission from Elsevier.

In 2011, Ji et al. used a dielectric/metal/dielectric multilayer as the top electrode, realizing the first top-emitting FWOLED with a maximum CE of 8.66 cd A^−1^ and CRI of 84 [222]. The key features for this FWOLED was the use of a conductive transparent cathode (i.e., MoO_3_ (40 nm)/Ag (17 nm)/MoO_3_ (40 nm)) to suppress the reflection of the metal layer and achieve a selective high transparent effect. The average transmittance of MoO_3_ (40 nm)/Ag (17 nm)/MoO_3_ (40 nm) in visible range was >84%, which was similar to ITO, as shown in Figure 6. In addition, the sheet resistance of MoO_3_ (40 nm)/Ag (17 nm)/MoO_3_ (40 nm) was very low (i.e., 11 Ω sq^−1^). By using the flexible PET substrate, the device structure was Al (100 nm)/MoO_3_ (1.5 nm)/4, 4′, 4″-tris(3-methylphenyl-phenylamino)-tripheny-lamine (m-MTDATA, 30 nm)/NPB (10 nm)/4,4′-bis(2,2′-diphenylethenyl)-1,1′-biphenyl (DPVBi, 15 nm)/CBP (3 nm)/CBP: bis(2-(2-fluorophenyl)-1,3-benzothiazolato-N,C2′)iridium acetylacetonate [(F-BT)_2_Ir(acac)] (7 nm)/4, 7-diphenyl -1, 10-phenanthroline (Bphen, 30 nm)/LiF (1 nm)/Al (1 nm)/Ag (1 nm)/MoO_3_ (40 nm)/Ag (17 nm)/MoO_3_ (40 nm), where Al/MoO3, m-MTDATA, NPB, DPVBi, CBP: (F-BT)_2_Ir(acac), Bphen, and LiF/Al/Ag/M/A/M were the anode, hole injection layer, hole transport layer, blue fluorescent EML, orange phosphorescent EML, electron transport layer, and cathode, respectively. The Al was used as the anode replacing the common Ag because Al film had larger phase shift on reflection than that of Ag, while the neat CBP was introduced to separate blue and orange EMLs to avoid Dexter energy transfer between the two emitters. As a result, a top-emitting FWOLED was organized. Later, Ji et al. also demonstrated that the multilayer electrode (Alq_3_/Ag)_2_ was promising for FWOLEDs [223].

### 3.4. FWOLEDs Based on Phosphorescent Emitters

#### 3.4.1. Phosphorescent FWOLEDs Employing a Single-Layer Graphene Anode

When electrons meet holes in OLEDs, singlet excitons and triplet excitons can be generated with the ratio of 1:3. To develop high-efficiency FWOLEDs, all generated excitons should be harvested. Hence, phosphorescent or TADF emitters are generally required, since they can harvest the triplet excitons. With the design of advanced device architecture, the maximum IQE of phosphorescent or TADF OLEDs will be unity. In addition, effective outcoupling technologies are needed to further enhance the efficiency. As a matter of fact, almost all reported high-performance FWOLEDs were established via the combination of phosphorescent/TADF emitters and outcoupling technologies.

**Figure 7:**
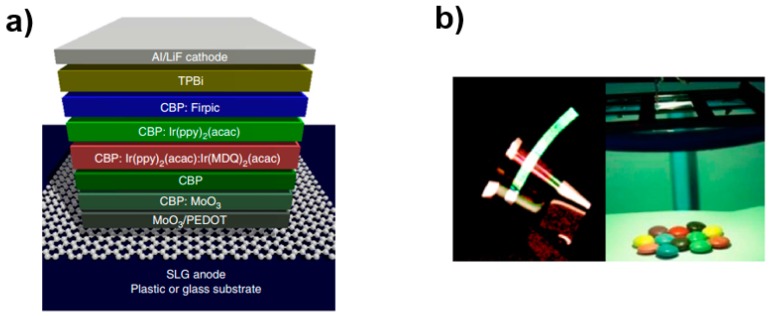
(**a**) The device structure of FWOLED, (**b**) photos of FWOLED and a high brightness FWOLED illuminating colored objects [224]. Copyright (2013) with permission from Springer Nature.

In 2013, Li et al. reported high-efficiency FWOLEDs by using a phosphorescent device structure and effective outcoupling technology (Figure 7), yielding an EQE of external quantum efficiency >45% at 10,000 cd m^−2^ (CRI = 85) and a PE of 80 lm W^−1^ at 3,000 cd m^−2^ [224]. In this work, single-layer graphene was used as the flexible transparent anode, which was different from Han’s FWOLED with four-layer graphene anode [221]. Since multiple-layer graphene required multiple graphene transfers (higher cost) and suffered from light absorption (each additional layer of graphene absorbs ~3% of light across the spectrum), the use of single-layer graphene would solve these issues. To ensure that holes could be efficiently injected into EML, a p-type chemical doping was performed by soaking the graphene sample in 1 mg ml^−1^ triethyloxonium hexachloroantimonate/dichloroethene solution, producing a charge transfer complex, leading to an increased work function (5.1 eV), an improved carrier density (2 × 10^13^ cm^−2^), and a decreased sheet resistance (<200 Ω/square). Furthermore, PEDOT:PSS/MoO_3_ was utilized as the interface layer to enhance the work function of the graphene anode to 6.7 eV, which was lower than the highest occupied molecular orbital (HOMO) of the CBP based EML (6.1 eV), remarkably enhancing the hole injection. Therefore, the key feature for Li’s FWOLED was the highly efficient hole injection from single-layer graphene to light-emitting layers, eliminating the efficiency roll-off due to carrier trapping, charge imbalance, and exciton quenching at the anode/organic interface. With a flexible PET substrate, the configuration was single-layer graphene anode/PEDOT:PSS/MoO_3_/CBP: MoO_3_ (hole injection layer)/CBP/CBP: bis[2-(2-pyridinyl-N)phenyl-C](acetylacetonato)iridium(III) [Ir(ppy)_2_(acac)]: bis(2-methyldibenzo[f,h]quinoxaline) (acetylacetonate) iridium (III) [Ir(MDQ)_2_(acac), red EML]/CBP: Ir(ppy)_2_(acac) (green EML)/CBP: Bis(4,6-difluorophenylpyridinato-N,C2)picolinatoiridium (Firpic, blue EML)/TPBi (electron transport layer)/LiF/Al (cathode). Then, the outcoupling method including substrates and lenses made of high index glass (n = 1.80) was used to further enhance the efficiency, achieving a maximum EQE of 51% and a PE of 90 lm W^−1^ at 1,000 cd m ^−2^. As a result, a high-performance FWOLED was developed.

#### 3.4.2. Phosphorescent FWOLEDs Utilizing a Simplified Outcoupling Approach

For Li’s FWOLED [224], the efficiency of the FWOLED was comparable to general lighting. However, the improvement in optical outcoupling that relied on high refractive index substrates was unfavorable to low cost mass production. Furthermore, half sphere lens are usually only suitable for small-area devices, which are not practical for large-area applications. Therefore, a simplified, low-cost but effective outcoupling approach with suitability for large-area applications is beneficial to free the light trapped by the substrate of FWOLEDs.

**Figure 8:**
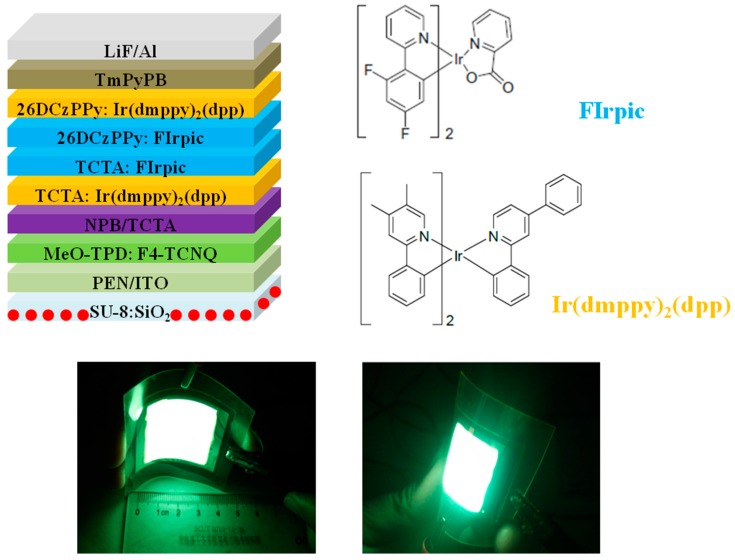
**Top**: The schematic structure of FWOLED and chemical structure of emitters. **Bottom**: Photographs of large-area FWOLED (30 mm × 30 mm) working at 1000 cd m^−2^ [225]. Copyright (2014) with permission from Royal Society of Chemistry.

In 2014, Liu et al. realized a FWOLED with an outcoupling film that was fabricated by dispersing SiO_2_ (n = ~1.5, average particle sizes of 1.5 μm) into SU-8 matrix with a concentration of 15%, exhibiting a maximum forward-viewing PE of 101.3 lm W^−1^ [225]. Furthermore, the device exhibited excellent color stability with a CIE variation of (0.004, 0.005) when the luminance increased from 100 to 10000 cd m^−2^. As shown in Figure 8, with the 120 μm PEN substrate, the device configuration was ITO (170 nm)/MeO-TPD: F4-TCNQ (100 nm, 4%)/NPB (15 nm)/TCTA (5 nm)/TCTA: Ir(dmppy)_2_(dpp) (1 nm, 20%)/TCTA: FIrpic (4 nm, 7%)/26DCzPPy: FIrpic (4 nm, 20%)/26DCzPPy: Ir(dmppy)_2_(dpp) (1 nm, 20%)/TmPyPB (50 nm)/LiF (1 nm)/Al (200 nm), where ITO was an anode, F4-TCNQ was tetrafluoro-tetracyanoqinodimethane, doped into N, N, N′,N′- tetrakis(4-methoxyphenyl)-benzidine (MeO-TPD), TCTA is 4,4′,4″-tri(9-carbazoyl) triphenylamine (an exciton/electron blocking layer and a host of orange/blue emitters), Ir(dmppy)_2_(dpp) was Bis(2-phenyl-4,5-dimethylpyridinato)[2-(biphenyl-3-yl)pyridinato] iridium(III) (an orange emitter), FIrpic was a blue emitter, 26DCzPPy was 2,6-bis(3-(carbazol-9-yl)phenyl)pyridine (a host of orange/blue emitters), TmPyPB was 1,3,5-tri(m-pyrid-3-yl-phenyl)benzene, LiF was an electron injection layer and Al was a cathode. For this structure, the main exciton generation zone was located at the TCTA/26DCzPPy interface, which was effectively broadened via the double blue EML. In addition, to stabilize the color, enhance the efficiency, and to reduce the efficiency roll-off, some device strategies were adopted, i.e., the optimized EML thickness, the orange EMLs designed to surround blue EMLs for harvesting the unused excitons, the charge transport layers of TCTA and TmPyPB having high S_1_ and T_1_ to confine singlets and triplets in the EML, and the reduction of charge mobility associated with host-dopant energy level difference. To further enhance the performance of FWOLEDs, an outcoupling approach was proposed by simply dispersing SiO_2_ into SU-8 matrix to fabricate scattering films, extracting the light trapped by the substrate. Therefore, the key features for the high performance could be summarized: (i) a multifunctional carrier- and exciton-confining structure was designed to guarantee the high efficiency and stable color; (ii) a simplified, low-cost but effective outcoupling approach with suitability for large-area applications was used to free the light trapped by the substrate.

#### 3.4.3. Phosphorescent FWOLEDs Having Plastic with Embedded Ag Network Anode

In Liu’s work [225], the efficiency of the FWOLED was demonstrated to exceed 100 lm W^−1^. However, in addition to the limited bendability, the use of an ITO anode cannot avoid the ITO/organic waveguide mode due to the high refractive index of ITO. As a result, optical loss may limit the further enhanced efficiency of such a FWOLED. To unlock the potential efficiency, high-quality transparent conductive electrodes with superior stretchability and roll-to-roll manufacturing compatibility to replace ITO is required, apart from the feasible outcoupling technologies. Based on this strategy, FWOLEDs are expected to possess much higher efficiency. 

**Figure 9:**
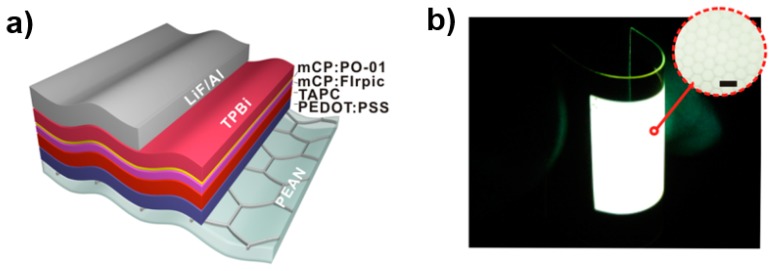
(**a**) Schematic of FWOLED device structure using plastic with embedded Ag networks as anode. (**b**) Photograph of a large-area FWOLED (50 mm × 50 mm). Inset: the magnified image taken with an optical microscope (scale bar = 3.00 μm) [226]. Copyright (2014) with permission from American Chemical Society.

By combining a transparent conductor on plastic with embedded silver networks as the anode and an outcoupling structure simultaneously extracting light in waveguide and substrate modes and reducing the surface plasmonic losses, Tang et al. presented a FWOLED showing a maximum EQE of 49% and PE of 118.1 lm W^−1^ [226]. For the flexible OLEDs using this new anode, only a small decrease in efficiency (19%) after 1000 bending cycles occurred. In addition, the new anode exhibited a low surface roughness to avert electrical short circuits and a lower sheet resistance in comparison with ITO at a given transparency. As shown in Figure 9, with the PET substrate, the device structure was the new anode/PEDOT:PSS/di[4-(N,N-ditolylamino)phenyl]cyclohexane (TAPC, 45 nm,hole transport layer)/N,N′-Dicarbazolyl-3,5-benzene (mCP): 8 wt % FIrpic (19 nm, blue EML)/mCP: 6 wt % iridium(III) bis(4-phenylthieno[3,2-c]pyridinato-N,C2′) acetylacetonate (PO-01, 1 nm, yellow EML)/TPBi (40 nm, electron transport layer)/LiF (1 nm)/Al (100 nm). The key features for the high light extraction efficiency were: (i) the well matched refractive index of plastic with embedded Ag networks (n = 1.49) to PEDOT:PSS (n = 1.46) and organic layers (n = 1.75), eliminating the photon flux trapped in the ITO/organic waveguide mode, enhancing the light from organic EMLs into the substrate; (ii) a light outcoupling structure by nanoimprinting the PEDOT:PSS layer with deterministic aperiodic nanostructures to promote the extraction of light originally trapped in the organic waveguide mode into the plastic substrate; and (iii) a microlens array applied to the plastic substrate to extract light from the substrate to the air. Therefore, a high-efficiency FWOLED has been developed. With the similar device architecture and a new metal-dielectric composite electrode, Tang et al. also reported a highly efficient FWOLED without outcoupling structure, exhibiting a maximum EQE of 47.2% and PE of 112.4 lm W^−1^ [227].

#### 3.4.4. Transparent Phosphorescent FWOLEDs Possessing Plastic with Embedded Ag Grid Anode

The motivation for using metal-dielectric composite electrodes is that they are effective in terms of mechanical flexibility, electrical conductivity, optical transparency, and large-area film uniformity. Particularly, plastic with embedded Ag grid anodes can minimize the microcavity effect in OLEDs, which is favorable for high color quality. Another significant application for FWOLEDs is the development of transparent devices. However, it is challenging to realize the high-performance transparent FWOLEDs, considering three crucial aspects are required to be simultaneously addressed, i.e., transparent electrodes, device architectures, and outcoupling methods.

**Figure 10:**
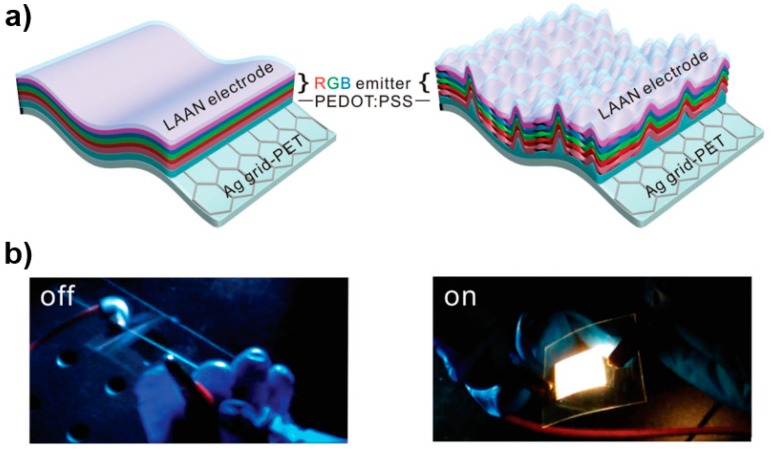
(**a**) Schematic of FWOLEDs on plastic without and with internal moth-eye outcoupling structure. (**b**) Photographs of a FWOLEDs in off and on states [93]. Copyright (2018) with permission from John Wiley and Sons publisher.

To unlock the great potential of transparent FWOLEDs, Tang et al. recently reported a device that could collectively reduce ohmic losses and release the trapped photons, achieving a maximum EQE of 72.4% and PE of 168.5 lm W^−1^ with a CRI of 84.5 [93]. For the bottom transparent electrode, an embedded silver grid with hexagonal structure on a PET substrate was employed, then 80 nm PEDOT:PSS was spin-coated onto this PET to promote hole injection into an organic stack with triple electrophosphorescent EMLs, as shown in Figure 10. Such metal-dielectric composite electrodes were excellent alternatives due to their high performance in terms of efficiency and mild angular dependence on emission spectra. In addition, the sheet resistance of this electrode was only 4.7 Ω sq^−1^, improving the electrical behaviors due to the reduced ohmic loss. On the other hand, the top transparent electrode was composed of 1 nm LiF electron-injection layer/1.5 nm Al seeding layer/15 nm Ag conductive layer/70 nm NPB optical coupling layer. Therefore, these two transparent electrodes ensured that the white emission could be emitted from both sides. With regards device architecture, an efficient organic emitter with negligible energy loss during electron-photon conversion was designed, i.e., the bottom electrode/PEDOT:PSS/TAPC (50 nm) as the hole transport layer/TCTA (5 nm) as the electron blocking layer/mCP: 6 wt % Ir(MDQ)_2_(acac) (7 nm) for red emission/mCP: 8 wt % Ir(ppy)_2_(acac) (3.5 nm) for green emission/mCP: 8 wt % FIrpic (5.5 nm) for blue emission/TmPyPB (60 nm) as the electron transport/hole blocking layer/the top electrode. To further increase the efficiency, an outcoupling approach was used to suppress the substrate mode by employing an additional external moth-eye structure. Therefore, a highly efficient transparent FWOLED was presented (the maximum PE/EQE were 103.9 lm W^−1^/43.7% and 64.6 lm W^−1^/28.7% for the bottom and top side, respectively). The key feature of this transparent FWOLED was the integration of bioinspired moth-eye nanostructures into the transparent electrodes, which enabled the broadband angle independent outcoupling enhancement of the waveguide light and suppressed the surface plasmonic loss at the metal/dielectric interface with no impact on electrical properties.

From the development of FWOLEDs, it can be easily noted that the innovation of flexible electrodes is a hot topic, which is expected to replace the well-known ITO electrode. In fact, significant progress has been made on flexible electrodes. Apart from the above mentioned flexible electrodes, many efficient electrodes have also been reported. For example, Koo et al. developed a bottom-emitting FWOLED by using a multilayered metal stack anode of Ni/Ag/Ni treated with oxygen plasma for 60 s, obtaining a maximum EQE of 5.85% [228]. Chen et al. reported a FWOLED by modifying the graphene anode surface with PSS to improve the air-stability/hole-injection ability and reduce the leakage current, achieving a maximum PE of 128.2 lm W^−1^ and EQE of 99.5% [229]. In brief, the future target for flexible electrodes is that they should have outstanding optical transparency/electrical conductivity/mechanical flexibility and be accessible to large-scale manufacturing with low cost.

## 4. Summary and Outlook

Since FWOLEDs are not only compatible with the technology of conventional WOLEDs based on rigid glass substrates but also undergo remarkable mechanical deformation, these excellent characteristics mean that FWOLEDs are very promising for next-generation displays and lighting. Nowadays, the efficiency of state-of-the-art FWOLEDs is comparable to that of the best traditional WOLEDs based on glass substrate and ITO anode. In this review, we have mainly focused on recent advances in FWOLEDs. Particularly, we have emphasized representative FWOLEDs based on fluorescent, hybrid, and phosphorescent emitters. The detailed performances for FWOLEDs have been described in Table 2.

After about 14 years of development, the performance of FWOLEDs has been enhanced step-by-step. In particular, their performance has been greatly boosted by the combination of phosphorescent device architectures and efficient outcoupling technologies. With the evolution of flexible transparent electrodes, the performance of FWOLEDs has been further improved. Although negligible attention has been paid to FWOLEDs based on TADF emitters thus far, it can be easily predicted that such types of FWOLEDs will show high performance via effective device architectures due to the 100% triplet-harvesting efficiency of TADF emitters, which is similar to phosphors [230,231,232]. To date, there are still many challenges hindering the development of commercial productions such as the efficiency, efficiency roll-off, angular color stability, cost, and particularly operational stability. For the issue of efficiency, since the theoretical efficiency limit of WOLEDs is 248 lm W^−1^, there is much room for FWOLEDs to be enhanced [233]. According to Equation (1), the EQE is determined by four factors. For the outcoupling schemes, although several effective methods have been reported to enhance the efficiency of FWOLEDs, there is still a long way to go regarding compatibility with large-area productions and stabilizing the angular colors [234,235,236]. To design good device architectures, the charge and exciton distribution should be carefully manipulated, which is beneficial to efficiency, efficiency roll-off, color stability, and lifetime [237,238,239,240].

For the issue of lifetime, it is a key factor in determining whether FWOLEDs can meet the demands of commercialization. Generally, a lifetime of ≥10,000 h at ≥1000 cd m^−2^ is required for the commercial applications of WOLEDs. However, it is worth pointing out that the working stability of FWOLEDs has been negligibly reported, which may be attributed to the short lifetime of FWOLEDs. For example, 5.2 h at an initial luminance of 1000 cd m^−2^ was obtained in Liu’s FWOLEDs [225]. One of the reasons for the poor lifetime is that it is still a huge obstacle to realize stable phosphorescent WOLEDs, since blue phosphors easily suffer chemical degradation during device operation. However, blue phosphors are required for the reported highly efficient FWOLEDs [93,224,225,226]. To alleviate this difficulty, the use of hybrid or TADF device structures may be helpful [241,242,243]. Another strategy to prolong the lifetime is the introduction of advanced flexible encapsulation techniques, since OLEDs are sensitive to moisture and oxygen. By reducing the water vapor transmission rate of the encapsulation (e.g., multilayers fabricated by Al_2_O_3_ and rapid SiO_2_ atomic layer deposition [244] and organic-inorganic multilayer structures [245]) to the ideal encapsulating barriers (10^−6^ g/m^2^/day) [246], much longer lifetime can be expected. To make FWOLEDs more competitive, researchers should pay more attention to lifetime. After solving the mentioned issues, the prospect for mass production of FWOLEDs will be bright, and the proposed solutions are also beneficial to the related optoelectronic fields (e.g., display, lighting, laser, solar cell, photodetector, thin film transistor, and sensor) [247,248,249,250,251]. 

## Figures and Tables

**Table 1:** Summarized performances for the commonly used flexible substrates.

Substrates	Thickness	Weight	Transparency	Surface	Flexibility	Thermal Durability	Cost
Metals	Thin	Heavy	Poor	Rough	Good	High	High
Glasses	Thin	Moderate	High	Moderate	Poor	High	High
Plastics	From thin to thick	Light	High	Smooth	Good	Low	Low

**Table 2:** Summarized performances for representative FWOLEDs.

WOLEDs ^a^	Year ^b^	V_on_^c^ (v)	EQE_max_/_1000_^d^ (%)	PE_max_/_1000_^e^ (lm W^−1^)	CE_max_/_1000_^f^ (cd A^−1^)	CIE ^g^	CRI ^h^
Ref. [77]	2005	4.7	4.0/-	4.3/-	11.3/-	(0.33, 0.33)	-
Ref. [218]	2010	4.0	3.2/-	6.5/-	-/-	(0.32, 0.34)	-
Ref. [221]	2012	~2.8	-/-	-/-	16.3/-	(0.32, 0.42)	-
Ref. [222]	2011	~4.4	-/-	-/-	8.66/-	(0.43, 0.39)	84
Ref. [224]	2013	-	51/-	90/90	120/-	-	85
Ref. [225]	2014	3.1	-/-	101.3/58.2	96.8/79.1	(0.32, 0.47)	52
Ref. [226]	2014	-	49/46.3	118.1/106	-/121.5	-	-
Ref. [93]	2018	-	72.4/69.4	168.5/142.5	-/-	-	84.5

^a^ Representative FWOLEDs. ^b^ The year for the reported FWOLEDs. ^c^ Turn-on voltage. ^d^ Peak external quantum efficiency (EQE)/EQE at 1000 cd m^−2^. ^e^ Peak power efficiency (PE)/PE at 1000 cd m^−2^. ^f^ Peak current efficiency (CE)/CE at 1000 cd m^−2^. ^g^ Commission International de L’Eclairage (CIE) coordinates at ~1000 cd m^−2^. ^h^ Peak color rendering index (CRI).
